# The diagnosis and treatment of adrenocortical carcinoma in pregnancy: a case report

**DOI:** 10.1186/s12884-020-2737-1

**Published:** 2020-01-21

**Authors:** Yuanli Zhang, Zeng Yuan, Chunping Qiu, Shuyi Li, Shiqian Zhang, Yan Fang

**Affiliations:** 1 Department of Obstetrics and Gynecology, Qilu Hospital, Shandong University, 107 Wenhuaxi Road, Ji’nan, Shandong 250012 People’s Republic of China; 2 Radiology Departments, Qilu Hospital, Shandong University, 107 Wenhuaxi Road, Ji’nan, Shandong 250012 People’s Republic of China

**Keywords:** Pregnancy, Adrenocortical carcinoma, Differential diagnosis, Case report

## Abstract

**Background:**

Pregnancy complicated with adrenocortical carcinoma (ACC) is a sporadic syndrome that is characterized by hypertension, uncontrolled hypokalemia, severe heart failure, premature delivery and other adverse effects. The clinical presentation of adrenocortical carcinoma is vague and nonspecific, it is challenging to identify complications of pregnancy with adrenocortical carcinoma. Here we present a case of adrenocortical carcinoma during pregnancy. We describe how to distinguish secondary hypertension from other conditions and the importance of timely detection and treatment of such patients.

**Case presentation:**

A 22-year-old woman 30 weeks pregnant was hospitalized with uncontrolled hypertension and hypokalemia. An ultrasound examination of the right adrenal gland revealed a large mass. She underwent transabdominal adrenalectomy, and histopathology from the sample removed revealed an adrenocortical carcinoma. Five days after surgery, the patient had a premature rupture of the fetal membranes and gave birth to a newborn girl via vaginal delivery at 32 weeks of gestation. The newborn was transferred to the neonatal pediatrics ward, and the woman started receiving chemotherapy.

**Conclusions:**

Pregnancy with adrenocortical carcinoma is a rare condition. This case alerts the obstetricians that analysis of hypertension, hypokalemia, the plasma level and circadian rhythm of plasma cortisol provides a strategy to diagnose adrenocortical carcinoma during pregnancy.

## Background

Adrenocortical carcinoma (ACC) is a rare condition with an estimated annual incidence of 1–2 per 1 million [1]. The prognosis is poor with a 5-year survival of approximately 30% [2]. In ACC, excessive cortisol inhibits the pituitary secretion of gonadotropin, and this could cause ovulation disorders, hypomenorrhea, and irregular periods or menopause in the majority of female patients [3]. ACC is rarely diagnosed during pregnancy, mostly reported as isolated cases [4]. The clinical presentation of adrenocortical carcinoma are vague and nonspecific, and for these reasons, it is challenging to identify ACC during pregnancy. Here we present a case of ACC during pregnancy, and we describe how to distinguish secondary hypertension from other conditions and the importance of timely detection and treatment of such patients.

## Case presentation

**Fig. 1:**
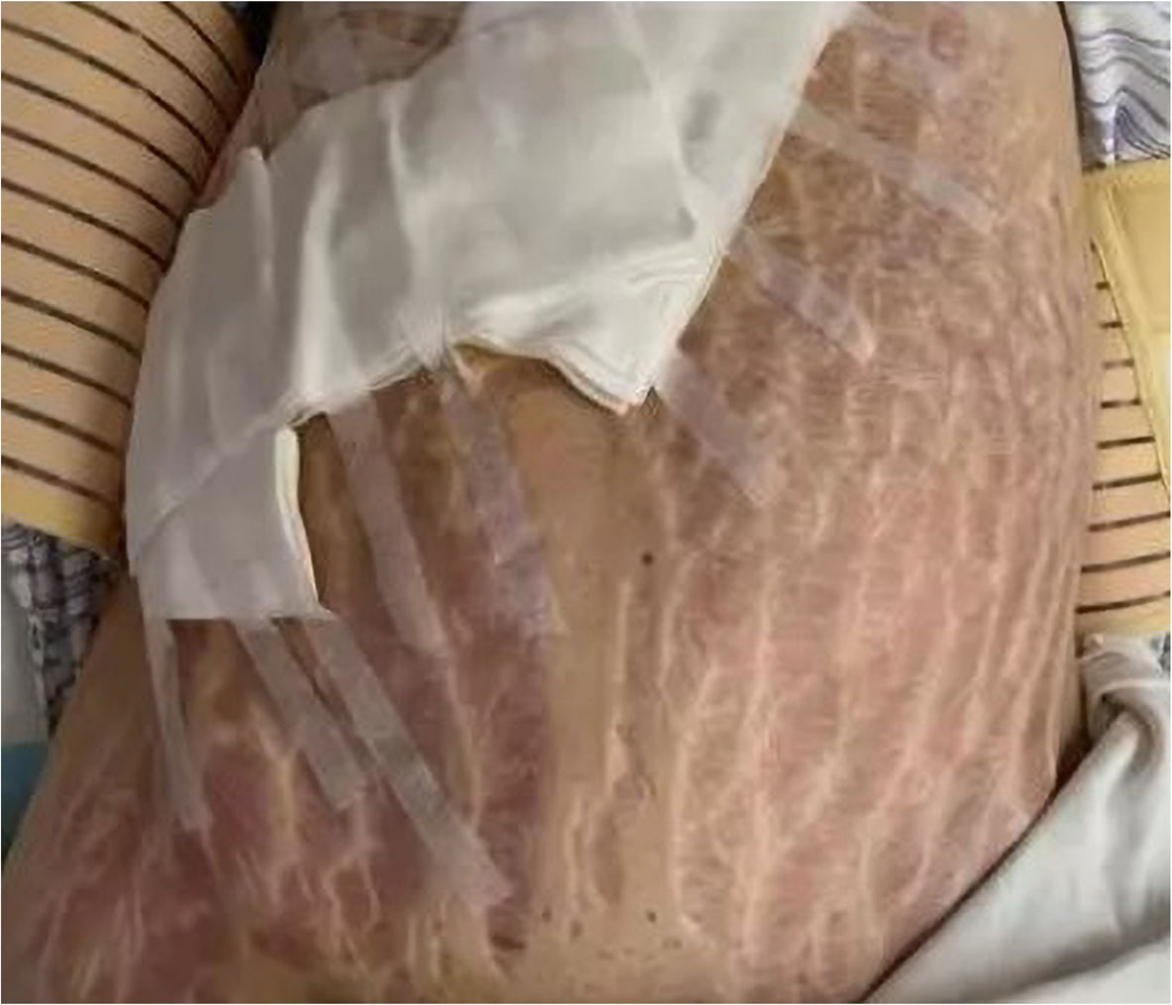
The patient’s lower abdominal skin was covered with broad, dark, thick striae (width is about 3.0 cm)

A 22-year-old woman with gestational age of 28 weeks and 4 days had a blood pressure of 161 /99 mmHg at a routine prenatal visit, and before that, her blood pressure was normal. The physical examination showed the following: Scattered ecchymosis in the neck skin, a purple ecchymosis of 5 × 4 cm size in the skin of the right forearm, and broad, dark, and thick striae in the skin of the lower abdomen (Fig. 1). The results of laboratory tests showed that concentrations of serum liver enzymes and creatinine were normal, suggesting that she did not have impaired liver function and renal insufficiency. Moreover, she did not have proteinuria. However, her serum potassium was 2.12 mmol/L, compared with non-pregnant values, serum potassium fell by 0.2–0.4 mmol/L, the rest of the serum electrolyte levels are in the normal range. After the administration of labetalol (300 mg, twice a day) and potassium, her blood pressure was remained in the range of 170 /100 mmHg and serum potassium maintained at 3.0–3.5 mmol/L. The electrocardiogram showed sinus tachycardia, while echocardiography indicated that her heart function was normal. Considering that the patient and her family had no previous history of hypertension and kidney disease, the liver and kidney function were normal, and the fetal ultrasound examination showed a fetus with a normal gestational age, the presence of uncontrolled hypertension and hypokalemia increased the suspicion that the problem was due to secondary hypertension rather than preeclampsia. The primary care physician ordered an ultrasound examination of the adrenal glands. The ultrasound showed a solid cystic mass (12.8 × 8.5 cm) in the right suprarenal gland region. Therefore, the patient was admitted to the endocrinology ward of Qilu Hospital of Shandong University with a diagnosis of “hypertension and hypokalemia of unknown origin” at 30 weeks gestation. Laboratory results showed the following values: glucose (−) in the urinalysis and her fasting blood glucose was 5.2 mmol/L, considering that she had no history of diabetes and had no polyuria and polydipsia recently, we ruled out the diagnosis of gestational diabetes. Plasma renin and aldosterone levels were in the normal range and the aldosterone/renin ratio (ARR) was 2.87 (normal range: 0–40). The 24-h urinary free cortisol level was 3357 μg/24 h (normal range: 21–110 μg/24 h), the serum cortisol level of 1246 nmol/L at 08:00 AM, 707 nmol/L at 04:00 PM (normal range: 112–288 nmol/L at 08:00 AM, < 128.7 nmol/L at 04:00 PM). The adrenocorticotropic hormone (ACTH) levels were 2.18 ng/L at 08:00 AM, 1.6 ng/L at 04:00 PM (normal range: 4.7–48.8 ng/L). The normal circadian rhythm of serum cortisol was lost, moreover, there was no significant inhibition in the low dose dexamethasone test. The above laboratory results supported a diagnosis of “ACTH-independent Cushing Syndrome,” therefore, a high dose dexamethasone test was requested.

**Fig. 2:**
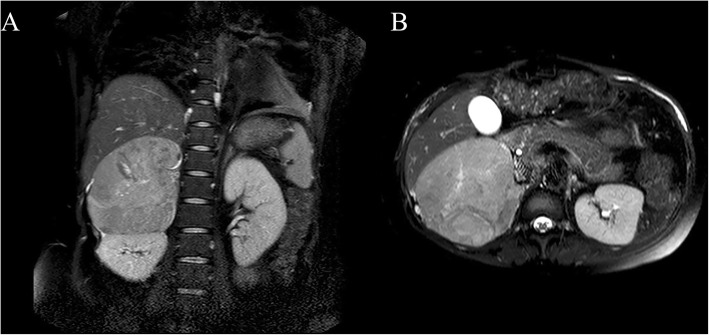
Coronal (**a**) and transverse (**b**) pelvic magnetic resonance imaging performed preoperatively demonstrates a soft tissue mass measuring 10.1 × 10.5 × 12.8 cm in the right retroperitoneal adrenal region, which oppressed the right kidney and liver

In order to localize the adrenal mass and its relationship with surrounding organs, we ordered an adrenal plain magnetic resonance imaging (MRI) without contrast agent 3 days after admission. The signal of the mass was slightly longer in T1 and T2-weighted image, and high signal intensity on diffusion-weighted imaging. In the T1-weighted image, multiple dot flake short signals were observed in the interior of the tumor, which had a size of approximately 10.1 × 10.5 × 12.8 cm. A short patchy hyperintensity in the T1-weighted image and a longer striate signal in the T2-weighted image were seen in the lesion. (Fig. 2). The above imaging features suggested the presence of hemorrhage in the lesion, and the location of the lesion was very close to the right adrenal gland. The imaging findings combined with the test results showing that a high dose of dexamethasone could not inhibit cortisol; therefore, we considered the possibility that an adrenal tumor was causing the Cushing syndrome.

The hospital held a case discussion board to obtain a consensus regarding the diagnosis and treatment to follow in this patient. Participants in this discussion board belong to various specialties such as obstetrics, urology, and anesthesiology, and they considered that hypertension and hypokalemia were caused by the mass in the adrenal region, and the symptoms would be relieved after the mass was removed. The discussion was focused on the ideal timing of treatment and the best surgical approach. The discussion board concluded that adrenalectomy should be performed by the urological department as soon as possible.

Transabdominal adrenalectomy was performed 12 days after admission. The abdominal cavity was opened using an inverted L-shaped incision 30 cm in length in the right lower abdomen. During the operation, a large mass (15x12x10 cm) was found in the right adrenal gland. The surface of this mass was hard in texture, and the surface envelope was intact and spread out all over the engorged blood vessels. The adhesions between the tumor and the superior, inferior vena cava, and surrounding tissues of the kidney was extreme. Also, the tumor squeezed the inferior vena cava, the right renal vein, and the right kidney, but it did not infiltrate the kidney and renal veins. The tumor were entirely resected during the operation and were sent to routine pathological examination. After tumor extraction, the urological surgeon closed the anterior sheath and placed a vacuum suction device subcutaneously, the skin at the wound was closed with skin staples. Postoperative day 1, ACTH increased to 78 ng/L, and plasma cortisol decreased to 172 nmol/L. Hydrocortisone was administered orally after the operation to prevent the occurrence of adrenal crisis. Unfortunately, the pathological results revealed that the tumoral mass was an ACC, and immunohistochemical staining of tissue specimens showed that: LH (−), HCG (−), and inhibin receptors (−).

Postoperative day 2, the patient was transferred to the maternity ward and treated with magnesium sulfate and ritodrine to inhibit the uterine contractions. At 32 weeks of gestation, she experienced a premature rupture of membranes. Considering her physical condition and disease state, we decided to stop using magnesium sulfate and ritodrine; therefore, her uterus naturally started contractions and entered the labor process. She had a vaginal delivery, the female newborn weighted 1360 g and had Apgar scores of 10 at both 1 and 5 min. Immediately after birth, the infant was transferred to the neonatology ward. Unfortunately, the newborn developed abdominal distension 2 days after birth, and there was no significant improvement after gastrointestinal decompression. Plain abdominal x-ray film showed intestinal perforation. The neonatologist suggested a surgical treatment, but the parents of the newborn refused. The newborn died 1 week after birth. The father of the newborn refused to perform an autopsy, thus, it was difficult to determine whether the death of the newborn was due to the impact of the maternal disease on the newborn or due to premature birth or other effects. Considering the patient’s condition, the oncology department recommended chemotherapy. Therefore, she was transferred to the oncology department for intravenous chemotherapy 3 days after delivery. After 6 months of follow-up, the hypertension and hypokalemia disappeared and she has completed chemotherapy at another hospital and without any clinical evidence of disease recurrence.

## Discussion and conclusions

In this case, the patient had elevated blood pressure at 28 weeks of gestation, so the main problem was differentiating the cause of hypertension. The four types of hypertension that are identified during pregnancy are gestational hypertension, preeclampsia or eclampsia, preeclampsia or eclampsia superimposed on chronic hypertension, and chronic hypertension (including primary and secondary hypertension). In 2019, the American College of Obstetricians and Gynecologists’ Committee (ACOG) defined gestational hypertension and pre-eclampsia as a systolic blood pressure ≥ 140 mmHg or a diastolic blood pressure ≥ 90 mmHg, or both, after 20 weeks of gestation, in a woman with a normal blood pressure previously [5]. However, preeclampsia is usually accompanied by new-onset proteinuria. Even if the patient’s protein is negative, other signs such as thrombocytopenia, renal insufficiency, impaired liver function, and pulmonary edema may occur. The patient had uncontrollable hypertension and hypokalemia, and blood pressure was not reduced after oral antihypertensive drugs, her liver function and renal function are normal, so it was not considered as preeclampsia. At the same time, the presence of elevated serum cortisol and wide gestational marks of the skin increased the suspicion that the problem was due to secondary hypertension.

Secondary hypertension during pregnancy includes chronic kidney disease, Pheochromocytoma, Primary aldosteronism, renovascular hypertension, Cushing’s Syndrome and obstructive sleep apnea causing hypertension. Pheochromocytoma and primary aldosteronism may also associated with severe hypokalemia. Persistent or paroxysmal hypertension is a typical symptom of pheochromocytoma, the diagnosis of it is established by measuring 24-h urinary fractionated metanephrines and catecholamines and plasma fractionated metanephrines. In addition to increasing aldosterone secretion, plasma renin activity is inhibited by increasing plasma aldosterone concentration in Primary aldosteronism. Given the adrenal mass with elevated plasma cortisol levels and the disappearance of circadian rhythm, we don’t think it is necessary to consider chronic kidney disease, pheochromocytoma, primary aldosteronism, renovascular hypertension as additional possible causes of hypertension and hypokalemia [6].

In a normal pregnancy, circadian cortisol rhythm still exists despite elevated cortisol levels. Currently, primary methods for diagnosing Cushing syndrome are 24-h free cortisol, midnight plasma or salivary cortisol levels, and low-dose dexamethasone suppression test (DSTs), as the HPA axis loses sensitivity to the effects of dexamethasone, more than 80% of DSTs in normal pregnant women produce false positive results [7]. Often, the diagnosis of pregnancy complicated with adrenal carcinoma is facilitated when the circadian rhythm of cortisol is lost [8]. Ultrasound and MRI are the recommended diagnostic imaging methods because both are safe for pregnant women and fetuses. Ultrasound is the preferred screening method, and if needed, MRI provides further diagnostic details [9]***.*** Although ACC screening largely depends on biochemical and imaging studies, and the final diagnosis depends on histopathology.

In general, ACC is characterized by an increase in serum sodium and a decrease in serum potassium, which is related to the glucocorticoid effect on sodium and potassium excretion. It has been reported that the decrease of serum potassium in patients with adrenocortical carcinoma is more evident than those in patients with suprarenal cortical adenoma [10]. This finding is also supported by the patient’s persistent and uncontrolled hypokalemia. Some researchers suggested that pregnancy is a risk factor for the deterioration of ACC [11]. Therefore, this disease should be considered when there are typical pregnancy symptoms complicated with refractory hypokalemia.

In patients with adrenocortical carcinoma during pregnancy, if the condition is not controlled, the harm to the fetus could be severe. Fassnacht et al. [12] reviewed the survival results of 416 patients from the German Adrenocortical Carcinoma Registry. They found that the patients’ prognosis was related to the stage of the disease at the time of diagnosis. When the tumor was limited to the adrenal gland and less than 5 cm, the 5-year disease-free survival rate was about 82%. When the tumor exceeded 5 cm but was still limited to the adrenal gland, the 5-year disease-free survival rate was close to 61%. If the disease extends beyond the adrenal gland, the survival rate declines. There are several clinical and in vitro tests that show a link between adrenal cortical proliferation and pregnancy. In an in vitro experiment, researchers found that increased expression of Aberrant GPCR expression in adrenocortical cells led to the formation of adrenocortical hyperplasia and the development of Cushing syndrome characteristics in transplanted mice [12]. Abiven-Lepage G et al. [2] compared 12 women diagnosed with ACC during pregnancy or immediately after delivery with non-pregnancy-diagnosed ACC patients, and found that patients diagnosed during pregnancy or postpartum had larger tumor volumes. However, the survival rate of patients diagnosed during pregnancy was 50% at 1 year and only 13% at 5 years. A retrospective single-center study showed that older age at diagnosis and cortisol hypersecretion were prognostic factor [13]. Data on fetal survival in pregnancy with ACC are unknown; reported cases could be biased toward the publication of successful pregnancy outcomes. Abiven-Lepage G et al. [14] reported that two newborns died (one abortion for medical reasons and one stillbirth) in 12 cases and five children were born before 37 weeks. Excessive steroid secretion in pregnant women with adrenal cortex could affect fetal sex differentiation, as shown in a case report of a 46 XX baby girl with ambiguous genitalia [15].

Surgical treatment is the first choice for patients during an early second trimester of pregnancy who have an apparent mass on the adrenal gland, medication is usually ineffective. It is relatively safe to perform surgery in the second trimester, any possible adverse events on the mother and fetus are minimal [16]. Unilateral or bilateral adrenalectomy has a good effect on alleviating hyper cortisol that is caused by an adrenal adenoma or adenocarcinoma and can also improve the perinatal outcome. After the operation, intravenous or oral administration of hydrocortisone is recommended to avoid adrenocortical insufficiency [17]. The laparoscopy approach is the preferred surgical technique due to the advantages of the short operation time, little interference to the abdominal cavity, and less bleeding. However, carbon dioxide (CO_2_) pneumoperitoneum during the operation could increase the blood CO_2_ partial pressure of the pregnant women, which might pose a potential threat to the fetus. Besides, the enlarged uterus could have an impact on the operation [18]. The tumor might not be completely removed through a laparoscopic approach considering the large volume of the adrenal gland. Therefore, we choose to perform transabdominal adrenalectomy.

In summary, ACC during pregnancy is sporadic and difficult to distinguish from other diseases that cause hypertension during pregnancy. Therefore, when hypertension and uncontrolled hypokalemia are present with adrenal masses, the possibility of secondary hypertension should be considered in the diagnosis, the analysis of plasma level and circadian rhythm of plasma cortisol provides a strategy to diagnose adrenocortical carcinoma during pregnancy.

## Data Availability

Datasets used and/or analyzed in the current study are available from the corresponding author by request.

## Abbreviations

ACC: Adrenocortical carcinoma
ACOG: The American College of Obstetricians and Gynecologists’ Committee
ACTH: Adrenocorticotropic hormone
CO2: Carbon dioxide
DSTs: Dexamethasone suppression test
MRI: Magnetic resonance imaging
